# Supplementary material for: Ribosome rescue factor PELOTA modulates translation start site choice and protein isoform levels of transcription factor C/EBPα
Source: bioRxiv. 2023 Jan 17:2023.01.16.524343. Preprint. [Version 1] doi: 10.1101/2023.01.16.524343 (PMC9882168; doi:10.1101/2023.01.16.524343)
Supplement: Supplement 2 [file NIHPP2023.01.16.524343v1-supplement-2.pdf]

bioRxiv preprint doi: https://doi.org/10.1101/2023.01.16.524343; this version posted January 17, 2023. The copyright holder for this preprint (which was not certified by peer review) is the author/funder, who has granted bioRxiv a license to display the preprint in perpetuity. It is made available under aCC-BY 4.0 International license.

Supplementary Figure 1

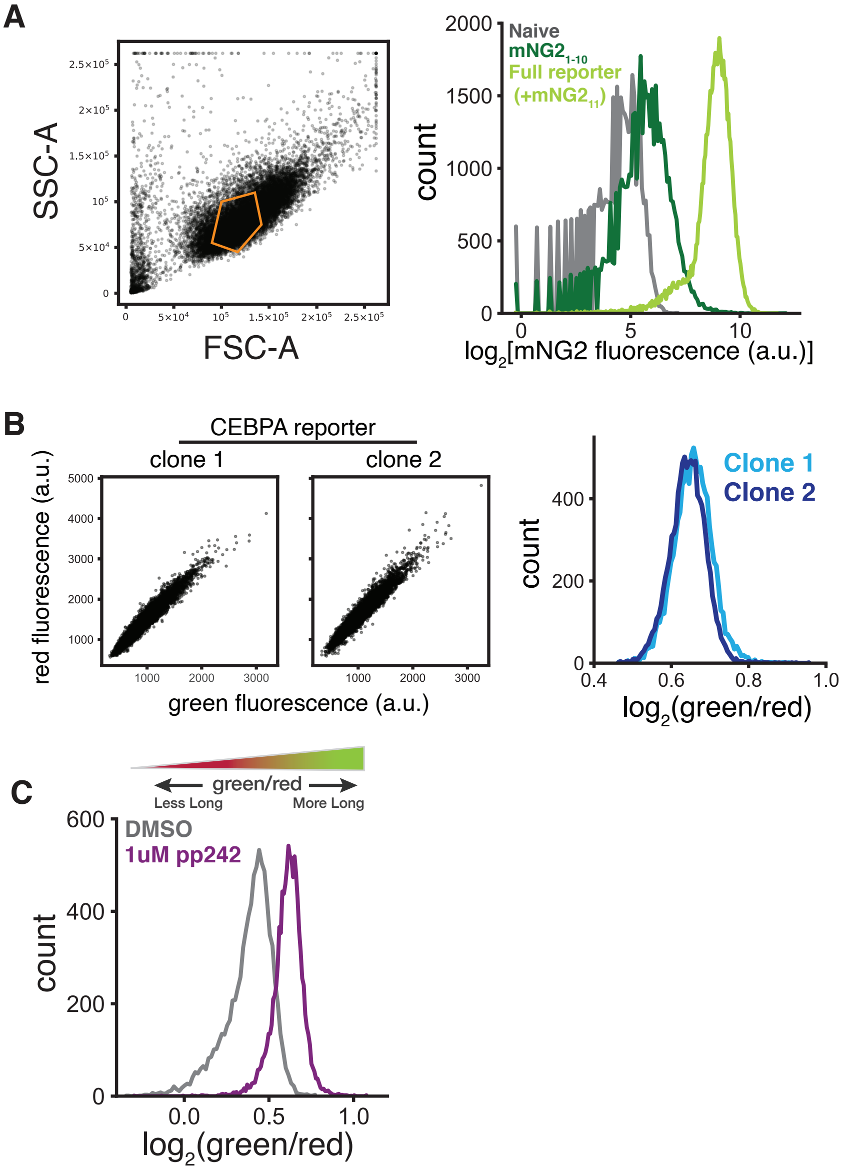

Supplementary Figure 1: Validation of *CEBPA* two color reporter (A) Forward (FSC-A) and side scatter (SSC-A) gating scheme for all flow cytometry measurements (left). Distribution of mNeonGreen2 (mNG2) fluorescence by flow cytometry from cell lines containing neither mNG2 fragment (naive), a cell line constitutively expressing only the mNG2_{1-10} fragment or a cell line expressing both mNG2_{1-10} and the *CEBPA* reporter containing the mNG2_{11} fragment (Full reporter) (right). (B) Scatterplots of green and red fluorescence from two clonal, stable *CEBPA* two color reporter cell lines (left) and green/red fluorescence distributions from these two clonal lines by flow cytometry (right). (C) Green/red fluorescence distributions in wild type two color reporter cell lines treated with either DMSO or 1 μM PP242 (Sigma-Aldrich). Cells were harvested and assayed by flow cytometry 24h post-treatment.

bioRxiv preprint doi: https://doi.org/10.1101/2023.01.16.524343; this version posted January 17, 2023. The copyright holder for this preprint (which was not certified by peer review) is the author/funder, who has granted bioRxiv a license to display the preprint in perpetuity. It is made available under aCC-BY 4.0 International license.

Supplementary Figure 2

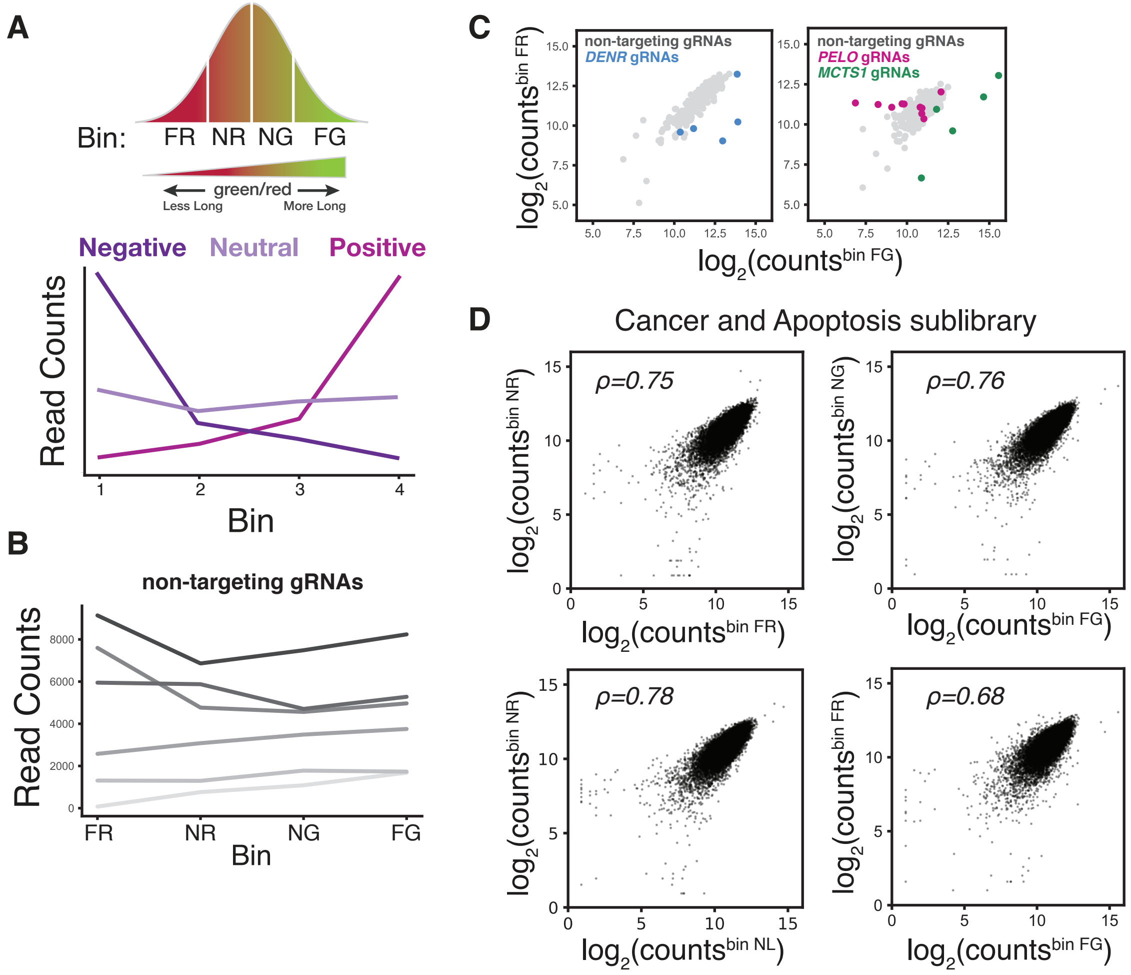

Supplementary Figure 2: Validation of FACS-based CRISPRi sublibrary screens (A) Schematic representation of possible, functional outcomes of sgRNA-mediated knockdown on the two color reporter. Bin labels reflect green/red distribution. FR: far red; NR: near red; NG: near green; FG: far green. sgRNAs that are enriched in cells with higher green/red ratios (NG and FG) may be changing the ratio by shifting expression towards the long isoform. We consider this class of sgRNAs as positive regulators of short isoform expression. In contrast, sgRNAs that are enriched in cells with lower green/red ratios (NR and FR) may be changing the ratio by shifting isoform expression towards the short isoform. We consider this class of sgRNAs as negative regulators of short isoform expression. sgRNAs that are uniformly distributed across all bins either have no effect on isoform usage or affect both isoforms equivalently. We classify these sgRNAs as being neutral. (B) sgRNA read count distribution across all bins for 6 nontargeting sgRNAs from the Gene Expression sublibrary. (C) Comparison of sgRNA read counts between the Far Red (FR) and Far Green (FG) bins in the Gene Expression sublibrary (left) and Cancer and Apoptosis sublibrary (right). Individual sgRNAs against *DENR*, *MCTS1* and *PELO* are highlighted. (D) Comparison of sgRNA read counts between indicated fluorescent bins in the Cancer and Apoptosis sublibrary (as in Figure 2D). Each point represents one distinct sgRNA.

bioRxiv preprint doi: https://doi.org/10.1101/2023.01.16.524343; this version posted January 17, 2023. The copyright holder for this preprint (which was not certified by peer review) is the author/funder, who has granted bioRxiv a license to display the preprint in perpetuity. It is made available under aCC-BY 4.0 International license.

Supplementary Figure 3

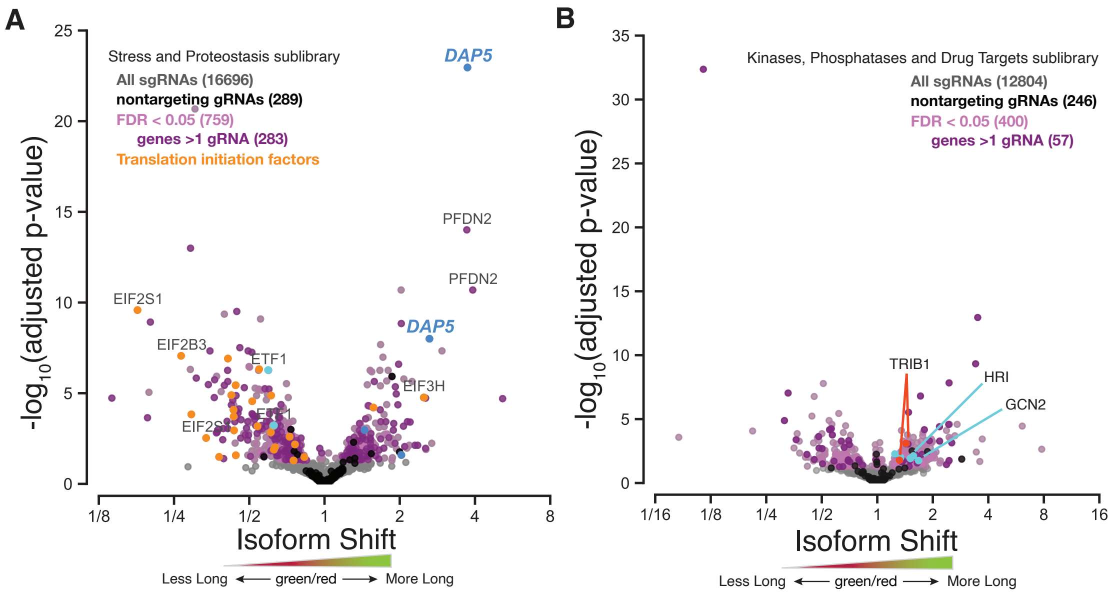

Supplementary Figure 3: Additional FACS-based CRISPRi sublibrary screen results (A) Stress and Proteostasis sgRNA sublibrary profile representing the relative shift in long and short isoform usage. Each point represents a single sgRNA with sgRNAs against *eIF4G2/DAP5*, translation termination factor 1 (*ETF1*) and translation initiation factors highlighted. Colors indicate cutoffs for significance (false discovery rate, FDR < 0.05), genes with at least 2 sgRNAs and nontargeting sgRNAs. (B) Kinases, Phosphatases and Drug Targets sgRNA sublibrary profile representing the relative shift in long and short isoform usage, as in (A). sgRNAs against the *eIF2α* kinases, *HRI* and *GCN2*, and the pseudokinase *TRIB1* are indicated.

bioRxiv preprint doi: https://doi.org/10.1101/2023.01.16.524343; this version posted January 17, 2023. The copyright holder for this preprint (which was not certified by peer review) is the author/funder, who has granted bioRxiv a license to display the preprint in perpetuity. It is made available under aCC-BY 4.0 International license.

Supplementary Figure 4

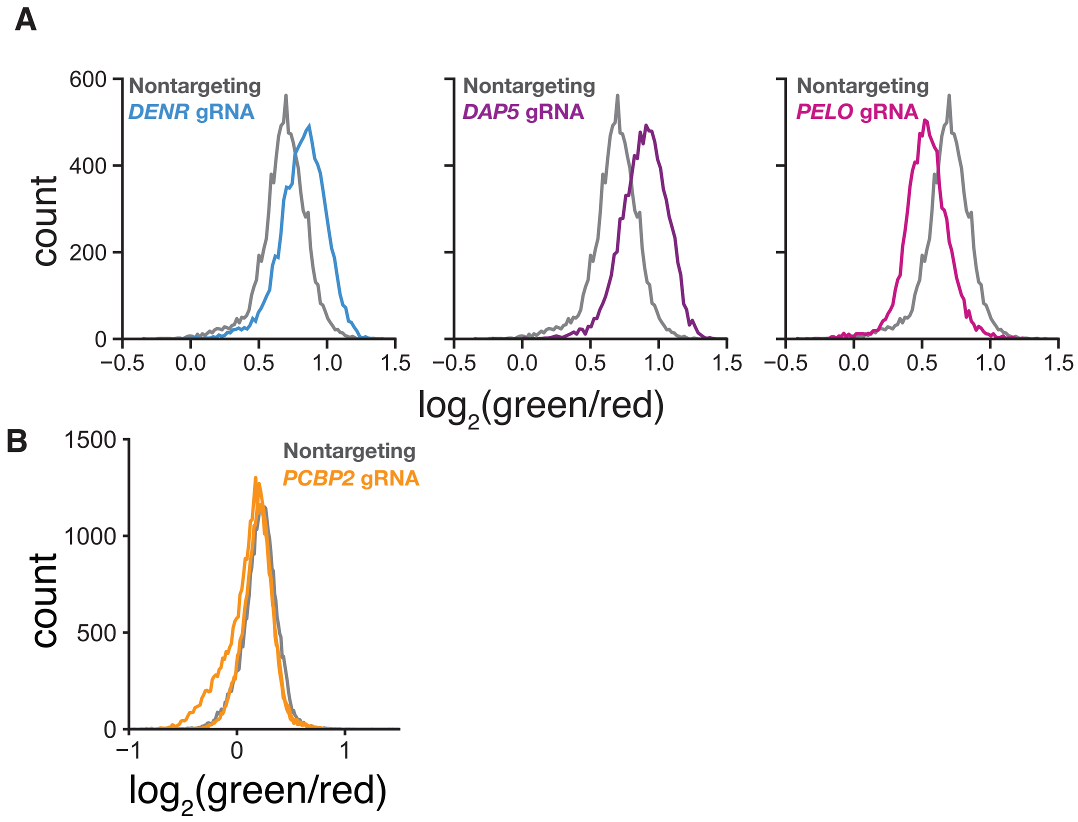

Supplementary Figure 4: Validation of individual sgRNA mediated knockdowns in two color reporter cell lines (A) Distribution of green/red fluorescence of a second, clonal two color reporter cell line transduced with the top scoring sgRNAs against either *DENR*, *DAP5* or *PELO*. (B) Distribution of green/red fluorescence of the two color reporter cell line transduced with 2 sgRNAs against *PCBP2*.

bioRxiv preprint doi: https://doi.org/10.1101/2023.01.16.524343; this version posted January 17, 2023. The copyright holder for this preprint (which was not certified by peer review) is the author/funder, who has granted bioRxiv a license to display the preprint in perpetuity. It is made available under aCC-BY 4.0 International license.

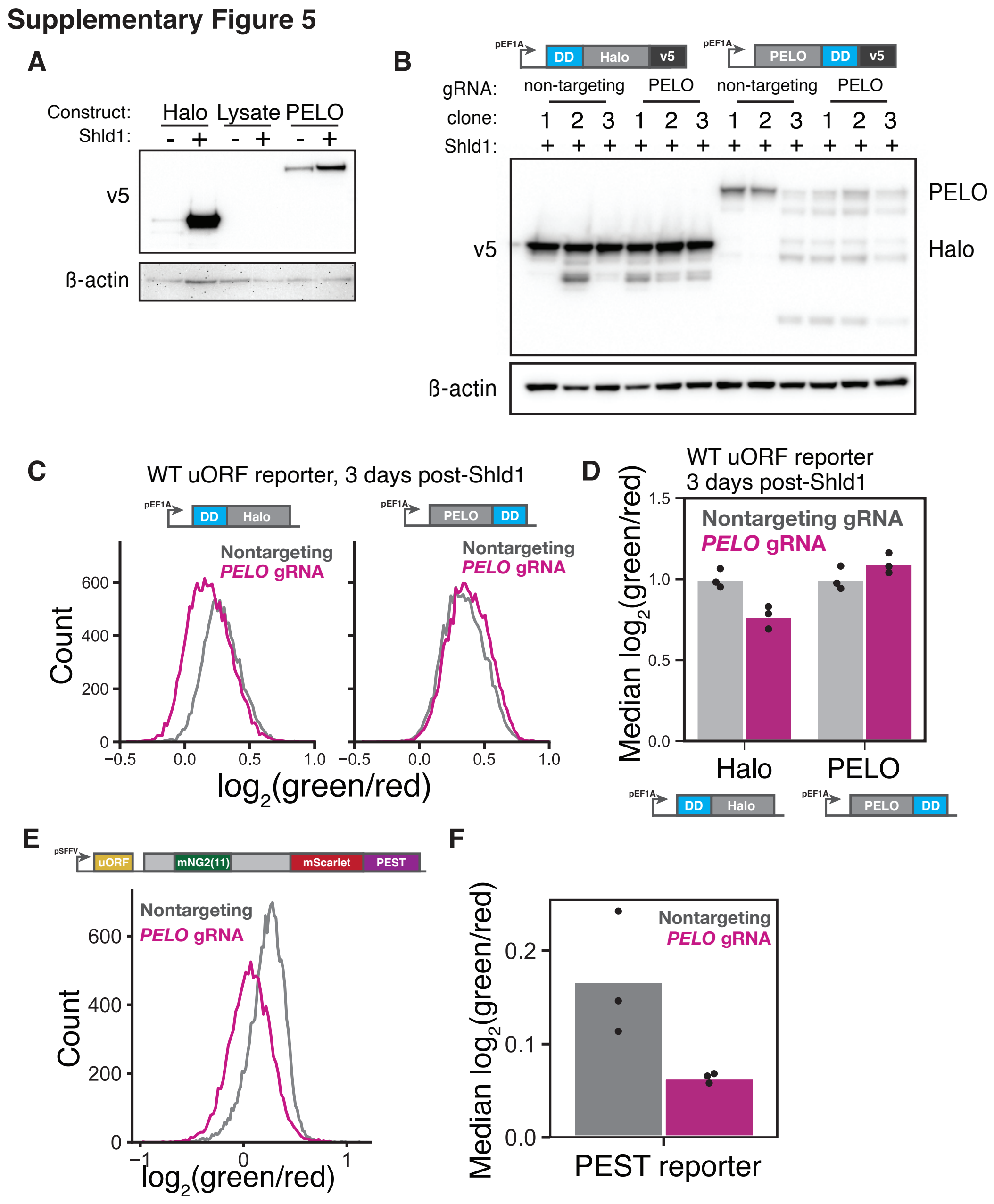

Supplementary Figure 5: *PELO* knockdown phenotype does not depend on uORF start codon context or differential protein stability (A) Western blot of naive K562 cells nucleofected with V5-tagged constructs containing either HaloTag or PELO fused to the FKBP12 destabilizing domain (DD) relative to β-actin loading control. Cells were either left untreated or treated with 1 μM Shield1 (Shld1) (Takara Bio) then harvested 72h post-treatment for Western blot. (B) Western blot of two color reporter cell lines stably expressing either a V5-tagged DD-HaloTag or PELO-DD fusion transduced with either a nontargeting sgRNA or a *PELO* sgRNA. β-actin was used as a loading control. DD-fusion cell lines were first transduced with sgRNAs, allowed to recover for 5 days then treated with 1 μM Shld1. Cells were harvested for Western blot 72h post-Shld1 treatment. Clones represent separate, independent transductions. (C) Distribution

bioRxiv preprint doi: https://doi.org/10.1101/2023.01.16.524343; this version posted January 17, 2023. The copyright holder for this preprint (which was not certified by peer review) is the author/funder, who has granted bioRxiv a license to display the preprint in perpetuity. It is made available under aCC-BY 4.0 International license.

of green/red fluorescence by flow cytometry in a two color reporter cell line harboring the native, uORF Kozak sequence (ctcgccATGc) stably expressing either a DD-HaloTag or PELO-DD fusion construct and transduced with either a nontargeting or a *PELO* sgRNA. Cells were transduced and treated as in (B). (D) Median green/red fluorescence measurements of the cell lines in (C), n = 3. (E) Distribution of green/red fluorescence by flow cytometry in a two color reporter cell line bearing a C-terminal PEST sequence transduced with either a nontargeting or *PELO* sgRNA. (F) Median green/red fluorescence measurements of PEST-fusion two color reporters in (E), n = 3.

bioRxiv preprint doi: https://doi.org/10.1101/2023.01.16.524343; this version posted January 17, 2023. The copyright holder for this preprint (which was not certified by peer review) is the author/funder, who has granted bioRxiv a license to display the preprint in perpetuity. It is made available under aCC-BY 4.0 International license.

Supplementary Figure 6

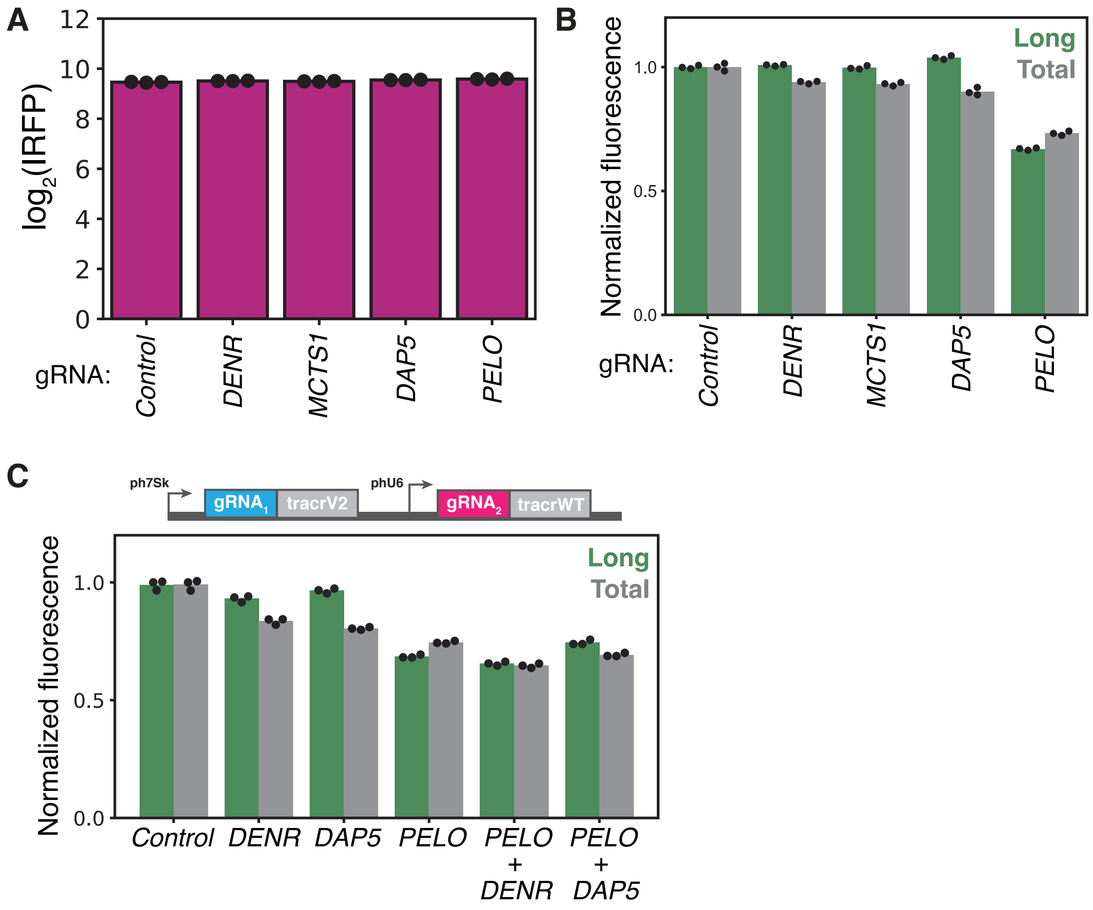

Supplementary Figure 6: Validation of *CEBPA* three color reporter (A) Flow cytometry measurements of median IRFP670 fluorescence in stable cell lines expressing the three color reporter transduced with indicated sgRNAs, n = 3. (B) Flow cytometry measurements of median green and red fluorescence normalized to IRFP670 in stable cell lines expressing the wild type three color reporter transduced with indicated sgRNAs, n = 3, as in Figure 5B. Fluorescent values were normalized to the median values in the negative, nontargeting control. (C) Flow cytometry measurements of median green and red fluorescence normalized to IRFP in stable cell lines expressing the wild type three color reporter transduced with indicated dual sgRNAs, n = 3, as in Figure 5C. Fluorescent measurements were normalized as in (B).

bioRxiv preprint doi: https://doi.org/10.1101/2023.01.16.524343; this version posted January 17, 2023. The copyright holder for this preprint (which was not certified by peer review) is the author/funder, who has granted bioRxiv a license to display the preprint in perpetuity. It is made available under aCC-BY 4.0 International license.

Supplementary Figure 7

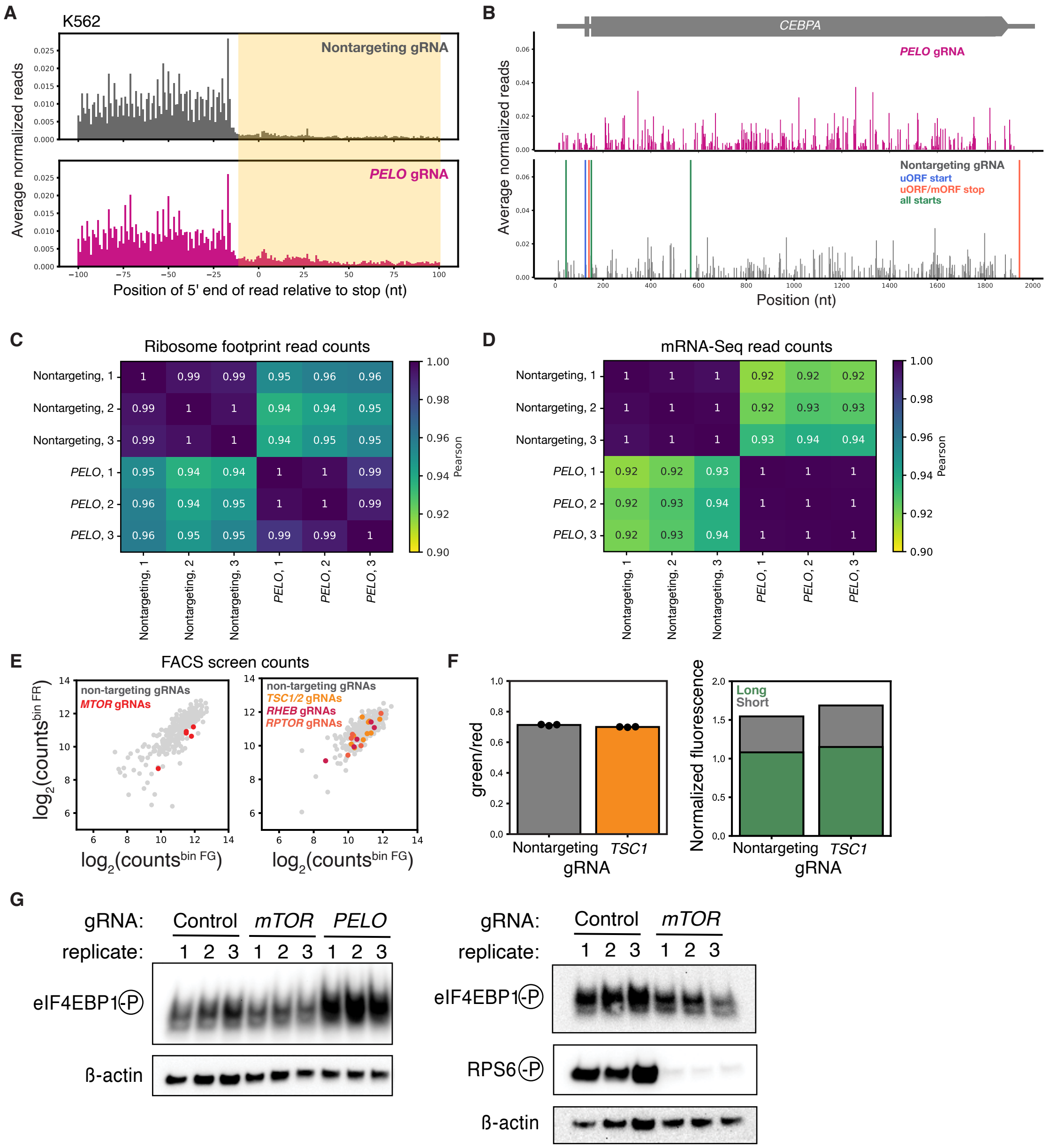

Supplementary Figure 7: *PELO* knockdown triggers mTOR activation (A) Metagene analyses of ribosome occupancy in either the nontargeting control or *PELO* knockdown in two color reporter line. Positions indicate the 5′ end of footprints relative to stop codons. Read counts were normalized by the total ribosome footprint count in each library and the median count was determined across replicates, n=3. (B) Ribosome footprint profile of the *CEBPA* reporter in cells expressing either a nontargeting sgRNA or a *PELO* sgRNA. Start and stop codons are indicated and counts were normalized as in (A). (C) Pearson correlation of ribosome footprint counts between indicated libraries. (D) Pearson correlation of ribosome profiling matched mRNA-seq counts between indicated libraries. (E) Comparison of sgRNA read counts between the Far Red (FR) and Far Green (FG) bins in the Kinases, Phosphatases and Drug Targets sublibrary (left) and

bioRxiv preprint doi: https://doi.org/10.1101/2023.01.16.524343; this version posted January 17, 2023. The copyright holder for this preprint (which was not certified by peer review) is the author/funder, who has granted bioRxiv a license to display the preprint in perpetuity. It is made available under aCC-BY 4.0 International license.

Cancer and Apoptosis sublibrary (right). Individual sgRNAs against *MTOR*, *RHEB*, *TSC1*, *TSC2* and *RAPTOR* are highlighted. (F) Flow cytometry measurement of three color reporter cell lines transduced with either a sgRNA against *TSC1* or a nontargeting sgRNA. Median green/red ratio is displayed, n = 3 (left). Flow cytometry measurements of median IRFP670 fluorescence in stable cell lines expressing the three color reporter transduced with indicated sgRNAs, n = 3 (right). (G) Western blot of three color reporter cell lines transduced with the indicated dual sgRNAs as in Figure 6D. β-actin was used as a loading control. Replicates represent separate, independent transductions.
